# Natural preservation strategies: The potential of bioactive compounds from fruit and vegetable waste to reduce postharvest losses

**DOI:** 10.1016/j.fochx.2025.102917

**Published:** 2025-08-13

**Authors:** Konstantinos Papoutsis, Zahra Shams, Mahboobeh Yazdani, Faraz Muneer, Mahbubjon Rahmatov, Nikwan Shariatipour, Evelyn Elizabeth Villanueva-Gutierrez, Monalisa Sahoo

**Affiliations:** a Department of Plant Breeding, Swedish University of Agricultural Sciences, Alnarp, Sweden; b Department of Horticultural Science, School of Agriculture, Shiraz University, Shiraz, Iran; c Departamento de Fitotecnia - Facultad de Ciencias Agrícolas y Pecuarias “Dr. Martin Cardenas”, Universidad Mayor de San Simón, P.O. Box 4894, Cochabamba, Bolivia

**Keywords:** Antifungal activities, Edible coatings, Horticultural waste, Natural extracts, Postharvest handling, Shelf-life, Waste valorization

## Abstract

The fruit and vegetable (F&V) waste generated postharvest has the potential of being used for the recovery of bioactive compounds that can be reintroduced into the supply chain to extend the postharvest quality of fresh produce. This review provides in-depth insights into the potential use of natural extracts derived from F&V waste to maintain the commercial and nutritional quality of fresh F&V. The mechanisms of action are comprehensively discussed. Application methods such as edible coatings, dipping solutions, and active packaging films are the main techniques used for the application of natural extracts. Future research integrating advanced -omics technologies is encouraged to unravel the molecular interactions of these bioactive compounds, facilitating the development of environmentally friendly, scalable postharvest treatments. Addressing regulatory challenges and ensuring industry adoption will be crucial in transitioning toward natural alternatives for food preservation, ultimately reducing postharvest losses and promoting sustainability in the fresh produce supply chain.

## Highlights

• Natural extracts affect fresh produce metabolism and physiology.
• Bioactives from f&v waste is a key opportunity for a sustainable agri-food system.
• Combined omics studies are essential for elucidating the mechanisms of action.
• Bioactive compounds from f&v waste can enhance food preservation and safety.
• Natural extracts may enhance fresh produce defense systems.

## Graphical abstract

**Figure:**
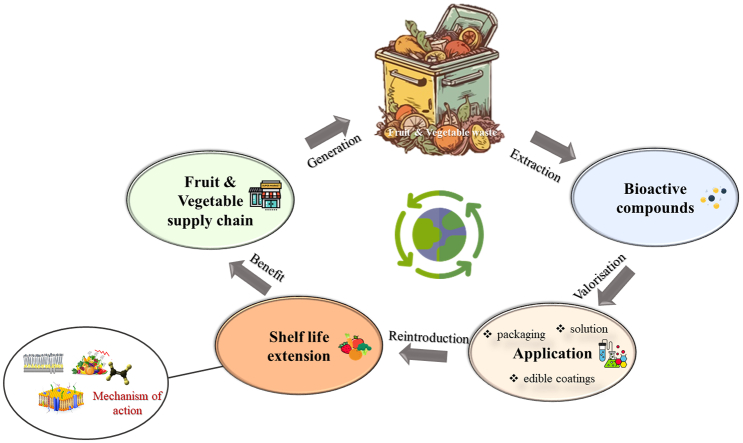

## 1 Introduction

**Fig. 1:**
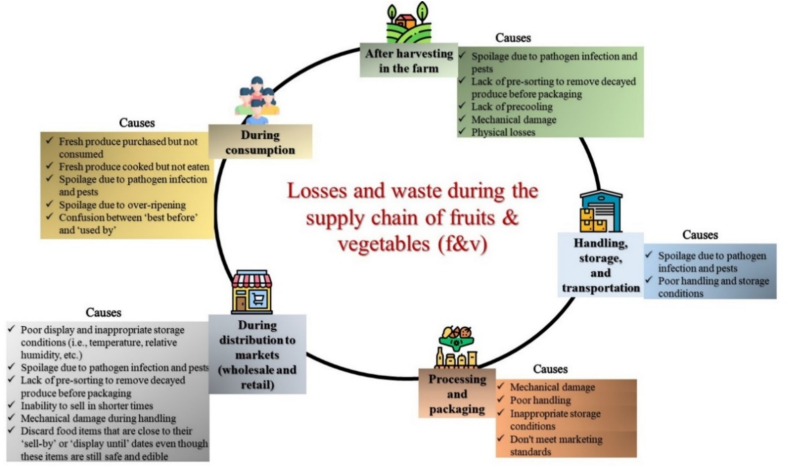
F&V losses from farm to fork according to Newson et al. (2025).

Fresh fruits and vegetables (F&V) are perishable with a short shelf-life due to their active metabolism and susceptibility to pathogen spoilage which can be accelerated due to inappropriate handling. It is estimated that up to 50 % of fresh F&V is lost from farm to fork globally (Bancal & Ray, 2022; Karoney et al., 2024). Fig. 1 presents specific causes that are responsible for fresh F&V losses and waste during the supply chain including i) farm after harvesting, ii) postharvest handling, storage, and transportation, iii) processing and packaging, iv) distribution to markets, and v) consumption (Newson et al., 2025). F&V loss and waste vary among the different countries based on their gross domestic product (GDP) per capita. For instance, in countries with higher GDP per capita, the highest food wastage takes place during food distribution and consumption. In contrast, in countries with lower GDP per capita, the highest food wastage takes place on the farm after harvest, during postharvest handling and storage (Chowdhury et al., 2025; Mak et al., 2020).

Synthetic pesticides are mainly used to extend fresh F&V postharvest quality. However, there is a growing public concern about the residues and toxicity of synthetic pesticides, which may pose risks to human health and the environment (Papoutsis & Edelenbos, 2021). Additionally, the European Union (EU) aims to reduce the risks and impacts of chemical use on human health and the environment by promoting the development of sustainable alternatives to synthetic pesticides (Directive 2009/128/EC). F&V waste also known as by-product, pomace or residue is also generated by the food industry after processing F&V for the production of juice, jam, canned food, etc. This type of waste is composed of peel fractions, pulps, and seeds, and represents approximately 16 % of the total food waste (Skwarek & Karwowska, 2023).

According to the Food and Agriculture Organization (FAO) of the United Nations (UN), the loss and waste of F&V represents a significant challenge with substantial consequences for global food security, social well-being, economic development, and environmental sustainability. A solution to this issue can be both the reduction and utilization of food losses and waste generated through all the stages of the supply chain (Chen et al., 2020). This is also included in the UN Sustainable Development Goals (SDGs) in the 2030 Agenda and especially in SDG 2 (Zero Hunger) and SDG 12 (Responsible consumption and production).

Fresh F&V contain a plethora of bioactive compounds with potential antioxidant, antifungal, and antimicrobial properties (Blejan et al., 2023; Papoutsis & Edelenbos, 2021). Therefore, F&V waste can be used as a raw material for the preparation of natural extracts that can be valorized by the food and horticultural industries as alternatives to synthetic pesticides and as functional materials for maintaining fresh F&V postharvest quality. Different application methods can be employed for the postharvest treatment of fresh F&V with natural extracts. Recently, a few review papers have been published, mainly presenting information about the potential application of plant-based extracts (derived from medicinal plants, *aloe vera*, lemongrass, neem, etc.) as alternatives to pesticides for preventing the postharvest development of pathogens (i.e., fungi) (Bajaj et al., 2023; El Khetabi et al., 2022; Nxumalo et al., 2021; Shahbaz et al., 2022). However, there is a knowledge gap related to the application and mechanism of action of extracts derived from F&V waste as environmentally and human-friendly postharvest treatments for extending the commercial and nutritional quality of fresh F&V. The aim of the current study is to present recent evidence regarding the potential postharvest application of extracts prepared from F&V waste as alternatives to synthetic pesticides to maintain fresh F&V postharvest quality. The mechanisms of action involved in the extension of fresh F&V treated with plant-based extracts are comprehensively discussed.

## 2 Bioactive compounds found in F&V waste and their extracts

**Fig. 2:**
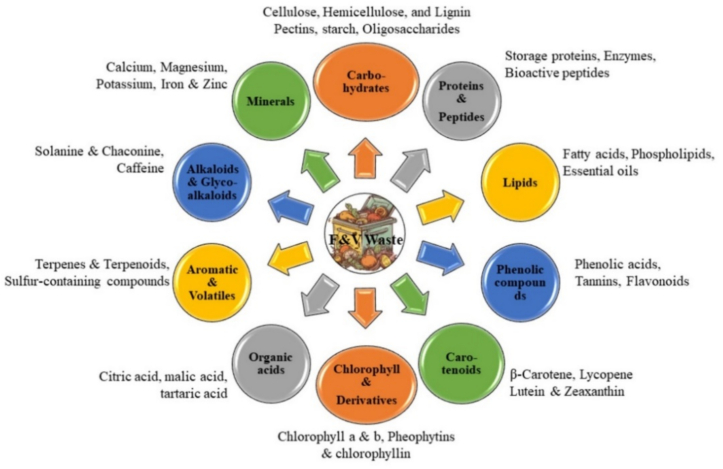
Different classes of compounds found in F&V waste.

The F&V waste mainly comprises peels, seeds, pomace, and leafy parts (i.e., leaves or stems). A wide array of phytochemicals are found in F&V waste including polyphenols, alkaloids, carotenoids, chlorophylls, fatty acids, coumarins, terpenoids, vitamins, minerals, enzymes, and essential oils, which have been linked to antioxidant, antifungal, antiviral, and antimicrobial properties (Fig. 2) (Byun et al., 2022; Papoutsis et al., 2018; Zhang et al., 2020; Zhao et al., 2017). During the last decade, several studies have been conducted aiming to valorize F&V waste for the extraction of bioactive compound(s) and their reintroduction to the food supply chain. The area of particular interest for their potential application is as food additives (to enhance nutritional deficiencies) and/or as alternatives to synthetic chemicals for preventing the development of pathogens (Maslov Bandić et al., 2025; Papoutsis et al., 2018; Rashid et al., 2022; Riaz et al., 2021). This is largely driven by increasing interest in the food and horticultural industry in replacing synthetic chemicals with natural compounds. The extraction of compounds of interest from F&V waste is highly sensitive to different parameters used during extraction e.g., types and parts of F&V used, pre-treatments used, and extraction methods and conditions (i.e., temperature and time, solvents) (Alara et al., 2021; Papoutsis et al., 2017). For applications in the food and horticultural industry, it is important to use environmentally and human-friendly non-toxic solvents when preparing extracts intended for postharvest application. Thus, developing regulations that specify suitable organic solvents for these extracts would support safer and more sustainable practices. Extracts from F&V waste can be applied either as crude extracts containing a broad spectrum of bioactive compounds or as fractions where specific bioactive compounds have been isolated. However, most existing studies have focused on crude extracts, making it challenging to pinpoint which compounds contribute to their beneficial properties. Additionally, a thorough investigation into food safety and toxicity is essential to ensure these extracts are safe for use as natural supplements and additives (Vilas-Boas et al., 2021). The following sections present information regarding studies that have used different types of F&V wastes as a source of valuable compounds that were applied to fresh F&V for extending their storage life and nutritional quality.

## 3 Methods for the application of F&V extracts

F&V waste extracts can be applied by i) incorporation into edible coatings, ii) as a solution where the F&V are immersed, and iii) incorporation into films used for packaging. Among the different methods, the incorporation of F&V extracts into edible coatings has been mainly reported (Table 1). The following subsections provide information about each method and their impact on F&V quality.

**Table 1:** Summary of different types of F&V wastes used for the preparation of extracts incorporated in edible membranes.

Source of extracts	Application method	Material edible coating based on	Fresh Produce	Storage condition/ self-life extension (days)	Quality parameters	References
Pomegranate peel extract	Dipping for N.M./ at N.M.	Xanthan gum-based	Mangos	22 °C and 60–65 % RH/ up to at least 12 days	• **Commercial and nutritional quality maintenance** • **Reduction of:** Weight loss, respiration rate, and ethylene production • **Maintenance of:** TSS, TA, pH, TPC, firmness, AA, antioxidant activity	(Kumar et al., 2023)
Cashew apple waste extracts	Dipping for 1 min/ drying for 24 h at room temperature	Xyloglucan- based	Guava	25 °C and 90–92 % RH/ up to 14 days	• **Commercial and nutritional quality maintenance** • **Reduction of:** Cell membrane lipid peroxidation, cell wall hydrolytic enzymes (i.e., PG and PME) activity • **Maintenance of:** Firmness • **Enhancement of:** Antioxidant enzyme activity (i.e., SOD, CAT, APX)	(Pontes et al., 2023)
Loquat seed starch	Dipping for 1 min/ drying for 24 h at room temperature	Loquat seed starch-based combined with glycerol or sorbitol	Strawberry	5 °C and 80 % RH/ up to 8 days	• **Commercial and nutritional quality maintenance** • **Reduction of:** Firmness and weight loss, microbial growth, pigment oxidation • **Maintenance of:** Visual and sensorial acceptance of the fruit	(Costa et al., 2023)
Pomegranate peel extract	Dipping/ air drying for 4–5 min at 30 °C	Taro starch-casein-based	Plums	5 or 23 °C and 50 % RH/ up to 20 and 14 days, respectively	• **Commercial and nutritional quality maintenance** • **Reduction of:** Firmness loss, respiration rate, TSS changes • **Maintenance of:** Antioxidant capacity, TPC, AA content	(More et al., 2022)
Mango peels and seeds	Dipping for 45 s/ drying at room temperature	Chitosan nanoparticles- based	Mango	28 °C and 96 % RH/ up to 10 days	• **Commercial quality maintenance** • **Reduction of**: Fungal infection incidence	(Istúriz-Zapata et al., 2022)
Mango peel extract	Dipping for 3 min/ drying for 10 min in a laminar air flow hood	Whey protein concentrate-based	Broccoli	5 °C and 80 % RH/ at least 21 days	• **Commercial and nutritional quality maintenance** • **Reduction of**: Respiration rate, AA loss, floret yellowing, weight loss, bacterial and fungal growth • **Maintenance of:** Color, antioxidant activity, and TPC	(Elsayed et al., 2022)
Pomegranate peel extract	Dipping for 5 min/ drying at room temperature	Chitosan nanoparticle- based and Selenium nanoparticle- based	Oranges (*Citrus sinensis*)	N.M. and 90 % RH/ up to 10 days	• **Commercial quality maintenance** • **Reduction of**: *Penicillium digitatum* growth	(Salem et al., 2022)
Pomegranate peel extract	Dipping for 2 min/ drying at room temperature	Chitosan-alginate-based	Tomatoes (*S. lycopersicum*)	20 °C and 60–70 % RH/ up to 21 days	• **Commercial and nutritional quality maintenance** • **Reduction of**: Firmness and weight loss, *Fusarium oxysporum* growth • **Maintenance of:** Acceptability, color, taste, and flavor	(Saeed et al., 2022)
*Carica papaya* seeds	Dipping for N.M./ dried for N.M.	Gum arabic- based	Tomato (*S. lycopersicum*)	Room temperature and N.M. RH/ up to 13 days	• **Commercial quality maintenance** • **Maintenance of:** Acceptability	(Kadirvel et al., 2022)
Radish leaf extract	Dipping for 2 min/ drying for 2 h at room temperature	Sodium alginate-based	Sweet lemon	4 °C and N.M. RH/ up to at least 40 days	• **Commercial and nutritional quality maintenance** • **Reduction of**: TSS changes, color changes, weight loss, and firmness changes	(Zandi et al., 2021)
Blackberry extract	Dipping for 5 min/ drying in an air circulation oven for 30 h at 35 °C	Carboxymethyl cellulose-based	Cherry tomato	6 °C and N.M. RH/ up to 15 days	• **Commercial quality maintenance** • **Reduction of:** Firmness and weight loss	(Sganzerla et al., 2021)
Orange peel oil extract	Dipping for 3 min/ drying at room temperature for 2 h	Carnauba wax-based	Strawberries	20 °C and 60–70 % RH/ up to 20 days	• **Commercial and nutritional quality maintenance** • **Reduction of:** Firmness and weight loss, *P. expansum* growth • **Maintenance of:** Acceptability, color, taste, and flavor	(Saeed et al., 2021)
Apple peel	Dipping for 30 s/ drying for 2 h at room temperature	Chitosan-based	Strawberries	20 °C and 35–40 % RH/ up to 6 days	• **Commercial and nutritional quality maintenance** • **Reduction of:** Weight loss, TSS changes, TA changes • **Maintenance of:** Firmness and acceptability, TPC, TFC, AA content, and total antioxidant activity	(Riaz et al., 2021)
Pomegranate peel extract	Brushing	Chitosan-based	Pears	0–1 °C and 90–95 % RH/ up to at least 60 days	• **Commercial quality maintenance** • **Reduction of:** Firmness and weight loss, membrane permeability increase, PG synthesis, PME synthesis, cellulase synthesis, and malondialdehyde synthesis	(Megha et al., 2021)
Pomegranate peel extract	Dipping for 2 min/ drying for 15 min using air dryer	Chitosan pullulan-based	Mangos	23 °C and 45 % RH or 4 °C and 95 % RH/ up to at least 15 days	• **Commercial and nutritional quality maintenance** • **Reduction of:** Weight loss, TSS changes, TA changes, antioxidant activity loss, TFC loss, and TPC loss • **Maintenance of:** pH and sensory characteristics (i.e., freshness, taste, color, and texture)	(Kumar, Pratibha, Neeraj, Petkoska, et al., 2021)
Pomegranate peel extract	Dipping for 2 min/ drying for 15 min	Chitosan pullulan-based	Bell peppers	23 °C and 40–45 % RH or cold 4 °C and 90–95 % RH/ up to at least 9 days	• **Commercial and nutritional quality maintenance** • **Reduction of:** Weight loss, TSS changes, TA changes, antioxidant activity loss, TFC loss, and TPC loss • **Maintenance of:** pH and sensory characteristics (i.e., freshness, taste, color, and texture)	(Kumar, Pratibha, Neeraj, Ojha, et al., 2021)
Pomegranate peel extract	Dipping for 2 min/ drying for 15 min at room temperature	Chitosan pullulan-based	Tomatoes	23 °C and N.M. RH or cold 4 °C and N.M. RH/ up to at least 9 days	• **Commercial and nutritional quality maintenance** • **Reduction of:** Weight loss, TSS changes, TA changes, antioxidant activity loss, TFC loss, and TPC loss • **Maintenance of:** pH and sensory characteristics (i.e., freshness, taste, color, and texture)	(Kumar, Neeraj, Pratibha, & Trajkovska Petkoska, 2021)
Pomegranate peel extract	Dipping for 3 min/ air-dried	Nanochitosan-based	Apricots	4 °C and 85–90 % RH/ up to 30 days	• **Commercial and nutritional quality maintenance** • **Reduction of:** Weight and firmness loss, antioxidant activity loss, AA loss, and decay • **Maintenance of:** Acceptability	(Gull et al., 2021)
Tomato pomace	Dipping for 5 min/ air-dried for 24 h	Locust bean gum-based	Tomatoes	4 °C and 85–90 % RH/ up to 28 days	• **Commercial and nutritional quality maintenance** • **Reduction of:** Weight loss, respiration rate, and decay	(Aloui et al., 2021)
Banana peel extract	Dipping for 1 min/ air-dried at room temperature for 30 min	Chitosan-based	Apples	25 °C and N.M. RH/ up to at least 20 days	• **Commercial and nutritional quality maintenance** • **Reduction of:** Respiration rate and weight loss • **Maintenance of:** Firmness, TSS, AA content, and TA	(Zhang et al., 2020)
Hairy Fig (*Ficus hirta Vahl.*) Fruit Extract	Dipping for 1 min/ dried at N.M.	Chitosan-based	Oranges	5 °C and 85–90 % RH/ for up to 120 days	• **Commercial and nutritional quality maintenance** • **Reduction of:** Weight loss, decay, respiration rate, and malondialdehyde content • **Maintenance of:** TSS, AA content, total sugar content, and TA • **Enhancement of:** SOD activity, POD activity, chitinase activity, and β-1,3-glucanase activity	(Chen et al., 2018)
Pomegranate peel extract	Dipping for 2 min/ dried at room temperature	Chitosan-based and Locust bean gum-based	Oranges	26 °C and 90 % RH/ for up to 5 days	• **Commercial quality maintenance** • **Reduction of:** Disease incidence caused by *P. digitatum*	(Kharchoufi et al., 2018)
Olive wastes extracts	Spraying/ air-dried at ambient temperature for 2 h	Chitosan-based	Strawberries	4 °C and N.M. RH/ for at least 8 days	• **Commercial and nutritional quality maintenance** • **Reduction of:** Decay, malondialdehyde content, antioxidant activity loss, TFC loss, and TPC loss	(Khalifa et al., 2016)
Grapefruit seed extract/ grapefruit oil	Dipping for 1 min/ air-dried for 24 h	Sodium alginate-based	Grapes	4 °C and 85–90 % RH/ up to 15 days	• **Commercial and nutritional quality maintenance** • **Reduction of:** Decay caused by *P. digitatum*	(Aloui et al., 2014)

AA: Ascorbic acid.

APX: Ascorbate peroxidase.

CAT: Catalase.

N.M.: Not mentioned.

PG: Polygalacturonase.

POD: Peroxidase.

RH: Relative humidity.

SOD: Superoxide dismutase.

TA: Titratable acidity.

TFC: Total flavonoid content.

TPC: Total phenolic content.

TSS: Total soluble solid content.

### 3.1 Edible coatings

Edible coatings are known as a simple and efficient method to prolong the shelf-life of fresh F&V (De Corato, 2020; Papoutsis, 2023). Edible coatings are composed of either one type of material such as polysaccharides (i.e., xanthan gum, locust bean gum, xyloglucan, loquat seed starch, chitosan, sodium alginate, carboxymethyl cellulose, and gum arabic), proteins (i.e., whey protein concentrate), and lipids (i.e., carnauba wax) or combined materials (also known as composite coatings) such as polysaccharide−polysaccharide (i.e., chitosan-pullulan) and polysaccharide−protein (taro starch-casein) (Firdous et al., 2023) (Table 1).

Several studies have noted that the efficiency of edible coatings to maintain the postharvest quality of fresh F&V can be improved with the addition of plant-based extracts enriched in bioactive compounds (Table 1). Plant-based extracts can be added to the solution of edible coatings in a liquid or powdery form (Chen et al., 2018; Elsayed et al., 2022; Saeed et al., 2022; Sganzerla et al., 2021). Edible coatings can be applied by spraying, dipping, and brushing. However, the most common method is dipping (Table 1). During application by dipping, fruits are dipped into a solution from 30 s to 5 min, and subsequently the treated fruits are left to dry from a few minutes to up to 24 h, depending on the drying method and temperature. Several parameters may affect the efficiency of the extracts incorporated into edible coatings to maintain the postharvest quality of fresh produce including type of the waste, extract concentration, interaction between extract and coating materials, and storage conditions (i.e., storage temperature and storage duration).

Wastes generated from different F&V (i.e., loquat seeds, cashew apple waste, pomegranate peel, mango peels and seeds, papaya seeds, orange peel, apple peel, banana peel, tomato pomace, olive waste, radish leaves, etc.) have been tested as a source for the preparation of extracts that can be subsequently incorporated into edible coatings (Table 1). During the last decade, edible coatings enriched in F&V waste extracts have been used to extend the postharvest quality and marketability of different fruits such as bananas, apples, strawberries, oranges, avocados, apricots, pears, mangos, sweet lemons, guavas, grapes, plums, bell peppers, and tomatoes and vegetables such as broccoli.

#### 3.1.1 Pomegranate waste extracts incorporated into edible coatings

Pomegranate peel extracts incorporated into edible films composed of different materials (i.e., chitosan-based, chitosan pullulan-based, chitosan alginate, starch-casein-based, xantham gum-based) have been shown to maintain the postharvest quality of various fresh produce including mangos, plums, oranges, tomatoes, pears, bell peppers, and apricots (Table 1). Specifically, the incorporation of pomegranate peel extracts incorporated into edible coatings can extend the marketability of fresh produce by acting as an antifungal agent and by retarding fresh produce metabolism. For instance, Salem et al. (2022) noted that pomegranate peel extracts enhanced the antifungal activity of nanochitosan-based coatings, thereby extending the storability of oranges by controlling the growth of green mold (*Penicillium digitatum*). Similarly, Saeed et al. (2022) reported that incorporating pomegranate peel extract enhanced the antifungal properties of a chitosan–alginate-based coating against *Fusarium oxysporum*, resulting in an extended storability of tomatoes for up to 21 days at 20 °C. Studies have also shown that incorporating pomegranate peel extracts into edible coatings can extend the commercial and nutritional quality of various fruits (e.g., mangoes, plums, oranges, tomatoes, pears, bell peppers, and apricots) by reducing respiration rates, ethylene production, and weight loss, while maintaining firmness, color, flavor, and the content of bioactive compounds and vitamins. For instance, Megha et al. (2021) noted that the combination of chitosan-based coating with pomegranate peel extract extended the postharvest quality of pears during 67 days of storage at 0–1 °C and 95 % relative humidity (RH) by maintaining firmness and reducing water loss. Similar effects of pomegranate peel extracts combined with chitosan-based coatings have been noted in tomatoes, bell peppers, apricots, and mangos. Similarly, Kumar et al. (2023) reported that xanthan gum-based coatings incorporated with pomegranate peel extracts maintained the commercial and nutritional quality of mangoes for at least 12 days at 22 °C and 65 % RH, by reducing respiration rates and ethylene production, and preserving fruit firmness, total soluble solids, titratable acidity, total phenolic content, and ascorbic acid levels.

#### 3.1.2 Mango waste incorporated into edible coatings

Mango waste extracts incorporated into edible films can extend the shelf-life of mangoes and broccoli. For instance, Elsayed et al. (2022) noted that the incorporation of mango peel extracts into whey protein-based coatings extended the quality of broccoli during 21 days of storage at 5 °C and 80 % RH by reducing floret yellowing, weight and ascorbic acid losses, while reducing the growth of bacteria. Mango waste extracts have the potential to be used in edible coatings as antifungal agents. The antifungal activity of the extracts varies among different fungi and it depends on the type of the waste and extract concentration. For instance, Istúriz-Zapata et al. (2022) reported that mango peel extracts exhibited stronger antifungal activity than mango seed extracts when incorporated into chitosan nanoparticle-based coatings. *In vitro*, the mango waste extracts reduced the mycelial growth of *Colletotrichum asianum* and *Talaromyces variabilis* in a concentration-dependent manner. *In vivo* experiments further demonstrated that disease severity and incidence were lower in mangoes coated with chitosan-based formulations containing mango waste extracts.

#### 3.1.3 Extracts from other F&V wastes incorporated into edible coatings

Pontes et al. (2023) noted that incorporating 1 % (*v*/v) cashew apple waste extracts into xyloglucan-based edible coatings effectively preserved the postharvest quality of guava during 14 days of storage at 25 °C and 92 % RH, notably maintaining fruit firmness and delaying ripening. Similarly, Chen et al. (2018) reported that incorporating hairy fig extract into a chitosan-based coating helped maintain the postharvest quality of navel oranges during 120 days of storage at 5 °C and 90 % RH by reducing weight loss and preserving total soluble solids, titratable acidity, sugars, and ascorbic acid content. Riaz et al. (2021) noted that polyphenols extracted from apple peels incorporated into chitosan-based films at a concentration of 1 % resulted in the extension of postharvest quality of strawberries during 6 days of storage at 35–40 % RH. The authors noted that chitosan-based coatings incorporated with apple peel polyphenols maintained quality parameters (i.e., weight, total soluble solids, titratable acidity, total phenols, total flavonoids, ascorbic acid content, total antioxidant activity) and delayed senescence of strawberries during storage. Khalifa et al. (2016) noted that that chitosan-based edible coatings enriched with olive waste extracts prolonged the commercial and nutritional quality of strawberries during cold storage at 4 °C for up to 8 days by reducing fruit decay and preserving total phenols, total flavonoids, and antioxidant activity. Zhang et al. (2020) showed that chitosan-based coatings incorporated with banana waste extracts effectively preserved the postharvest quality of apples during storage at 25 °C for up to 20 days by minimizing weight loss and maintaining fruit firmness, total soluble solids, titratable acidity, and ascorbic acid content.

#### 3.1.4 Negative impact of F&V waste extracts incorporated into edible coatings on fresh produce

Apart from the benefits of incorporating natural extracts into edible coatings, several challenges and drawbacks should be considered. For example, extracts rich in pigments such as anthocyanins and carotenoids can negatively affect the quality of fresh produce, particularly appearance, color, and flavor, when used at high concentrations. For instance, Sganzerla et al. (2021) noted that that edible coatings containing blackberry anthocyanin extracts at 10 %, 30 %, and 50 % concentrations adversely impacted the purchase appeal of cherry tomatoes by affecting color, overall appearance, brightness, aroma, and flavor. Therefore, future research should focus on optimizing extract concentrations in coatings, taking into account both physiological effects and quality parameters like appearance, color, and flavor. Incorporating oil extracts into coatings may accelerate water loss and firmness reduction by compromising the coating's water barrier properties and promoting senescence and cell wall breakdown. Citrus extracts are promising additives to extend the shelf-life of F&V, with oil- and polyphenol-based extracts both enhancing antifungal properties (Aloui et al., 2014; Saeed et al., 2021). However, the coating composition must be carefully chosen when incorporating citrus oils, as inappropriate combinations can harm postharvest quality. Saeed et al. (2021) that a carnauba wax-based coating with orange peel oil preserved strawberry quality over 20 days at 20 °C and 70 % RH by delaying firmness loss, weight reduction, and maintaining overall acceptability. Conversely, Aloui et al. (2014) noted that grapefruit essential oils in alginate coatings negatively affected strawberries stored at 4 °C and 90 % RH for 15 days, increasing water loss and reducing firmness. Future studies should aim to optimize coating formulations enriched with citrus extracts, considering the interactions between coating materials and extract types.

### 3.2 Immersion/dipping

In addition to their incorporation into edible coatings, extracts derived from F&V waste can also be directly applied as solutions, offering a simple and practical method for preserving fresh produce. Over the past decade, F&V waste extracts in solution form have been employed to extend the shelf-life of various fruits, including strawberries, oranges, clementines, apples, lemons, cherry tomatoes, and sweet cherries. These extracts help to maintain postharvest quality by reducing the incidence of spoilage due to pathogens (e.g., fungi and bacteria) and preserving the physicochemical and textural properties of the produce. Typically, fresh F&V are immersed in extract solutions prepared by dissolving the bioactive compounds in solvents such as water or ethanol. Treatment durations range from 30 s to 3 min, and application temperatures generally vary between 5 °C and 20 °C (Table 2). The type of solvent and the concentration of the extract are critical parameters that influence the effectiveness of the treatment in extending shelf-life.

**Table 2:** Summary of studies that used different types of F&V wastes for the preparation of extracts used either as an immersion treatment or incorporated into films used for packaging.

Source of extracts	Application treatment	Fresh Produce	Storage condition/ self-life extension (days)	Quality parameters	References
Coconut by-product aqueous extracts	Solution in artificially wounded area/ dried for 30 min at room temperature	Persian limes	15 or 25 °C and N.M. RH/ up to 13 days	• **Commercial quality maintenance** • **Reduction of:** Decay caused by *Penicillium italicum*	(Hernández-Flores et al., 2023)
Broccoli residue (leaves) extracts	Immersion for 2 min dried using paper towels	Cherry tomato	21 °C and 70 % RH/ up to 36 days	• **Commercial and nutritional quality maintenance** • **Reduction of:** Weight loss • **Maintenance of:** Firmness, color, pH, TSS, TA, and TA/ TSS ratio, TPC, antioxidant capacity	(Damas-Job et al., 2023)
Persimmon peel extract	Packaging	Banana	Storage temperature and RH N.M./ 7 days of storage	• **Commercial and nutritional quality maintenance** • **Reduction of:** Firmness and water loss, PPO activity, cellulase activity, PG activity, PME activity	(Hu et al., 2022)
*Allium* extract	Immersion for 30 s	Lemon	5 °C and N.M. RH/ 20 days of storage	• **Commercial quality maintenance** • **Reduction of:** Sour rot (*Geotrichum citri-aurantii*) incidence and the severity	(Fernández et al., 2022)
Pomegranate peel extract	Immersion for 3 min at 20 °C/ air-dried at room temperature	Sweet cherry	4 °C and 85 % RH/ up to 45 days	• **Commercial and nutritional quality maintenance** • **Reduction of:** Firmness and weight loss, respiration rate, decay • **Maintenance of:** TA, TSS, AA content, TPC, antioxidant activity, total anthocyanin content	(Parsa et al., 2021)
Pomegranate peel extract	Immersion for 30 s at 5 °C/ air-dried at 40 °C for 1 min	Oranges Clementines	4 °C and 95 % RH for 7 days followed by 7 days of storage at 20 °C	• **Commercial quality maintenance** • **Reduction of:** Decay caused by fungi, bacteria colonies	(Pangallo et al., 2021)
Pomegranate peel extract	Solution in artificially wounded area	Apple	20 °C 95 % RH/ at least 15 days	• **Commercial quality maintenance** • **Reduction of:** *Monilinia* spp. incidence • **Inhibition of:** Mycelial growth and spore germination	(El Khetabi et al., 2020)
Pomegranate peel extract	Immersion for 30 s at N.M. °C/ air dried for 1 h	Strawberries	4 °C and 60 % RH/ up to 7 days	• **Commercial quality maintenance** • **Reduction of:** *Botrytis cinerea* incidence	(Rongai et al., 2018)
Pomegranate peel extract	Immersion for 2 min /air dried for 2 h at room temperature	Sweet cherries Lemons	Lemons: 6 °C for 60 days of storage followed by 7 days of shelf-life at 20 °C Sweet cherries: 1 °C for 7 days followed by 3 days of shelf-life at 20–22 °C	• **Commercial quality maintenance** • **Reduction of:** *M. laxa* and *B. cinerea* incidence	(Li Destri Nicosia et al., 2016)

AA: Ascorbic acid.

N.M.: Not mentioned.

RH: Relative humidity.

PME: Pectin methylesterase.

PPO: Polyphenol oxidase.

TA: Titratable acidity.

TPC: Total phenolic content.

TSS: Total soluble solid content.

#### 3.2.1 Immersion into solutions prepared with pomegranate waste extracts

As shown in Table 2, pomegranate peel extracts have been widely investigated as postharvest treatments, primarily as alternatives to synthetic pesticides. These extracts have demonstrated strong antifungal activity, effectively controlling the growth of pathogens such as *Botrytis cinerea* and *Monilinia* spp., which are major contributors to F&V decay and postharvest losses. For instance, Pangallo et al. (2021) noted that applying pomegranate peel extract solutions significantly reduced decay in oranges and clementines. Similarly, other studies have shown that these extracts can preserve the quality of strawberries and sweet cherries during cold storage by reducing infections caused by *B. cinerea* and *M. laxa*, respectively (Li Destri Nicosia et al., 2016; Rongai et al., 2018)*.* The efficacy of pomegranate waste extracts can be further enhanced when combined with calcium salts. Specifically, Parsa et al. (2021) noted that combining 1 % CaSO₄ with 400 ppm aqueous pomegranate peel extract was more effective than when treatments applied individually in extending the shelf-life of sweet cherries stored at 4 °C and 85 % RH for up to 45 days. This combined treatment reduced weight and firmness loss, lowered respiration rate, and helped maintain titratable acidity, total soluble solids, ascorbic acid, total phenolics, antioxidant activity, and total anthocyanin content.

#### 3.2.2 Immersion into solutions prepared with other F&V waste extracts

In addition to pomegranate waste extracts, extracts derived from other F&V by-products (i.e., coconut residues and broccoli leaves) have also been evaluated as postharvest treatments to maintain the commercial and nutritional quality of fresh produce (Table 2). Hernández-Flores et al. (2023) noted that the postharvest application extracts from coconut by-products extended the commercial quality of Persian limes by reducing decay caused by *P. italicum*. Similarly, Damas-Job et al. (2023) compared methanolic and ethanolic broccoli leaf extracts and found that methanolic extracts (20 mg·L^−1^) were more effective in preserving cherry tomatoes stored for up to 36 days at 21 °C and 70 % RH. These extracts reduced weight loss and helped maintain texture, color, chemical attributes (pH, total soluble solids, titratable acidity, and their ratio), phenolic content, and antioxidant capacity. However, according to EU regulations, extraction solvents must be non-toxic and safe for human health. Methanol, while effective as a solvent, is a toxic alcohol that poses serious health risks, including morbidity and mortality upon exposure (Ashurst & Nappe, 2023). As a result, the EU has established a maximum methanol residue limit of 10 mg/kg in extracted foodstuffs or food ingredients (Directive 2009/32/EC). Therefore, methanol should be avoided when preparing natural extracts intended for use as postharvest treatments of F&V. Future studies should also prioritize evaluating the safety and toxicity of extraction solvents to ensure compliance with food safety regulations and to support the development of safe postharvest applications.

### 3.3 Incorporation into film used for packaging as active agent

Fresh F&V are living organisms with an active metabolism that continues even after harvest during storage at a wholesale, retail, and consumer level. F&V postharvest metabolism is affected by storage conditions such as temperature and atmosphere composition (i.e., CO_2_, O_2_, and ethylene (C_2_H_4_) levels) (Papoutsis, 2023). These atmospheric factors affect fresh produce respiration and induce quality changes in fresh produce (Ghidelli & Pérez-Gago, 2018; Papoutsis, 2023). Food packaging plays a critical role in protecting fresh produce from direct exposure to environmental conditions. The choice of packaging material significantly impacts the quality and shelf-life of packaged F&V. Traditionally, fresh produce is packaged in sealed polymeric films (Ghidelli & Pérez-Gago, 2018). However, increasing consumer and industry demand has driven the development of biodegradable and edible packaging materials that are non-toxic and more sustainable alternatives to synthetic polymers (Yadav et al., 2021). Recent studies have demonstrated that incorporating F&V waste extracts into edible packaging can enhance film functionality. For example, Hu et al. (2022) reported that the addition of 10 % persimmon peel extract to chitosan film significantly improved its physical properties and antioxidant activity. When applied to banana packaging, this functional film maintained fruit quality over 7 days of storage at 25 °C and 40 % RH. Bananas packaged with the enriched film exhibited greater firmness and fewer senescent spots compared to the control. The ability of the functional films to maintain bananas postharvest quality was attributed to the lower oxygen permeability of the films enriched with the extracts compared to the control. More research is encouraged to investigate the development of functional packaging films for extending the postharvest quality of F&V and elucidate the mechanism of action of the extracts incorporated into the films.

## 4 Mechanisms of action of the natural extracts

**Fig. 3:**
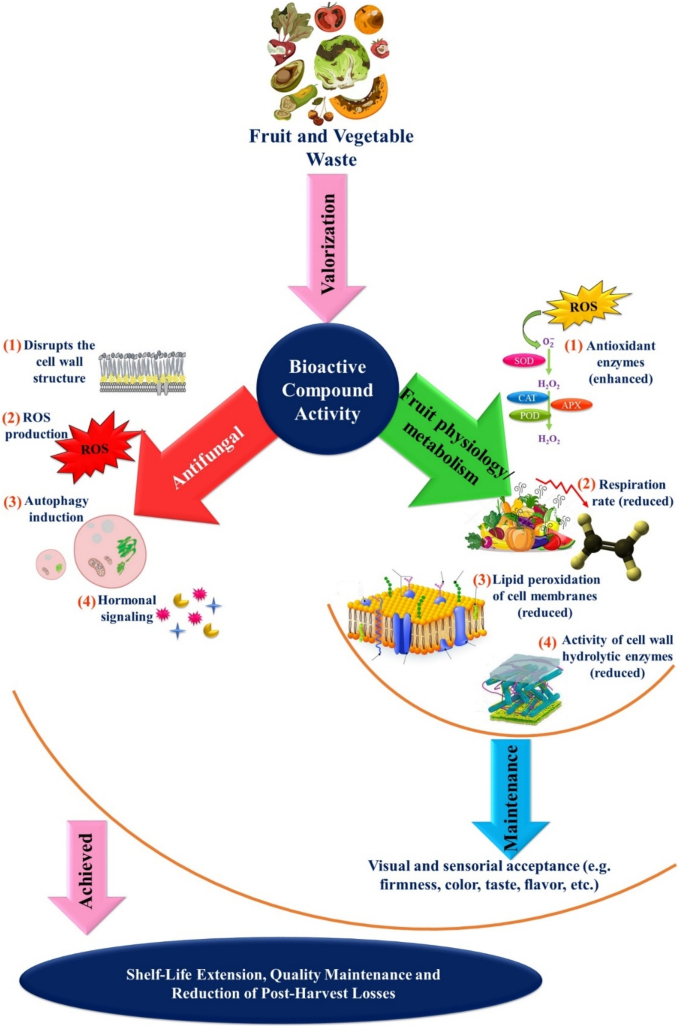
Potential mechanisms of action of extracts from F&V waste.

Natural extracts derived from F&V waste have recently attracted significant attention for their potential to enhance food preservation by reducing pathogen-induced decay while maintaining the nutritional and commercial quality of fresh produce. Natural extracts are considered Generally Recognized As Safe (GRAS), offering a sustainable solution to extend the marketability of fresh produce (Papoutsis et al., 2019). Natural extracts (also known as crude extracts) from F&V waste contain a diverse array of bioactive compounds that function through multiple mechanisms, including the suppression of produce metabolism, inhibition of pathogen growth, and mitigation of oxidative stress (Fig. 3). Gaining a deeper understanding of how these bioactive compounds exert their effects at a biochemical level is essential for optimizing their postharvest application to extend the storage of fresh F&V and reduce postharvest losses and waste. The following sections examine the potential mechanisms by which various treatments incorporating extracts from F&V waste contribute to maintain the postharvest quality of fresh produce, focusing specifically on the reduction of decay caused by pathogens (Section 4.1) and the preservation of key quality attributes such as firmness, color, and taste (Section 4.2).

### 4.1 Reduction of postharvest decay caused by pathogens (i.e., fungi)

Postharvest decay of F&V caused by pathogens is a major challenge in agriculture, leading to significant losses in the fresh produce supply chain from farm to fork, which can be up to 50 %. Among the different pathogens, fungi have the highest contribution to these losses (Youssef et al., 2023). Postharvest application of extracts from F&V waste have proven effective for preventing or delaying the growth of fungi (Table 1, Table 2). Research on the antifungal properties of plant-based bioactive compounds is still in its early stages, preliminary studies suggest that extracts of F&V waste may act through multiple mechanisms to counter fungal activity. Natural extracts may have direct effects on fungal metabolism and physiology and indirect effects by enhancing fresh produce defense systems (Fig. 3).

The direct effects of extracts include alterations in the cell structure of fungi, inhibition of cell division, disruption of electron transfer, inhibition of spore germination, induction of oxidative stress and autophagy, and finally fungal cell death (Fig. 3). Studies have shown that that incorporating pomegranate waste extracts into edible coatings enhances their antifungal activity against *P. digitatum* and *F. oxysporum*, thereby extending the storage life of oranges and tomatoes, respectively (Saeed et al., 2022; Salem et al., 2022). Comparable findings have been reported in other postharvest studies. The application of pomegranate peel extract solutions successfully inhibited the growth of *B. cinerea* and *M. laxa* in strawberries, sweet cherries, and lemons. Similarly, the use of coconut by-product extracts demonstrated effective antifungal activity against *P. italicum* in Persian limes (Hernández-Flores et al., 2023). Polyphenols from F&V waste extracts may exhibit strong antifungal activities through multiple mechanisms. For instance, they disrupt fungal cell structure, damage cell walls, and rupture hyphae. Punicalagin and tannins polyphenols found in pomegranate peel extracts exhibit antifungal activities by altering the cell structure of fungi, resulting in irregular cell walls and ruptured hyphae (Anibal et al., 2013; Salem et al., 2022). Phenolic acids can interact with membrane enzymes resulting in the loss of integrity of hyphae cell walls and the prevention of fungal growth (Fig. 3). Polyphenols may also inhibit spore germination by interfering with respiratory enzymes, and can induce autophagy and apoptosis, ultimately leading to fungal cell death (Chen et al., 2023; Papoutsis et al., 2018). Autophagy is a cellular degradation process that provides nutrients to eukaryotic organisms including fungi and plays an important role in fungal pathogenicity (Zhu et al., 2019). When autophagy is retarded results in the accumulation of reactive oxygen species (ROS), which causes an insufficient use of nutrients leading to cell wall collapse, while induction of autophagic activity and apoptosis results in the suppression of fungal growth and death (Chen et al., 2023; Liu, Cai, et al., 2021). More research is required to investigate the effects of different classes of bioactive compounds found in F&V waste extracts on fungal autophagic activities. Lipophilic compounds found in F&V waste extracts (i.e., essential oils) may target the fungal membranes causing leakage of cellular contents and inhibiting fungal growth (da Rocha Neto et al., 2019; Wang et al., 2020). Bioactive compounds found in F&V waste extracts may also induce the accumulation of ROS by inhibiting the mitochondrial electron transport chain, leading to oxidative stress and fungal cell death (Chen et al., 2023; Shang et al., 2021).

In addition to their direct antifungal effects, bioactive compounds in F&V waste extracts can enhance host resistance by activating the plant's innate defense systems. These indirect effects include accumulation of antioxidants and antifungal compounds, and synthesis of pathogenesis-related proteins (Fig. 3). Fungal invasion can cause the induction of ROS in the host plant (Segal & Wilson, 2018). Therefore, the enhancement of the antioxidant system in fresh produce can deactivate and scavenge the excess ROS, protecting plant tissues from pathogen-induced damage. For instance, compounds such as cinnamic acid and luteolin have been shown to induce the antioxidant system in F&V increasing fresh produce resistance to fungal infections and decay (Liu, Cui, et al., 2021; Zhang et al., 2015). Specifically, luteolin has been shown to reduce fungal decay by inducing the phenylpropanoid pathway, which leads to the synthesis of different classes of polyphenols, which are compounds with antifungal and antioxidant activities. Luteolin has also been found to induce the synthesis of antioxidant enzymes (i.e., superoxide dismutase (SOD), etc.) in fresh produce (Liu, Cui, et al., 2021). Similarly, cinnamic acid has been found to induce the phenylpropanoid pathway in treated plants (Zhang et al., 2015). Phytohormones like salicylic acid and jasmonic acid play key roles in enhancing plant defense by modulating signaling pathways that increase pathogen resistance (Jiao et al., 2018; Majeed et al., 2024; Segarra et al., 2006). Plant extracts may contain these phytohormones or stimulate their production in treated fruits. For example, salicylic and jasmonic acids have been detected in crude extracts of *Cucumis sativus* (Segarra et al., 2006). Future studies should investigate how bioactive compounds in F&V waste extracts regulate plant phytohormone pathways to prevent fresh produce decay. Bioactive compounds found in F&V waste extracts can also prevent fungal decay by stimulating the production of pathogenesis-related proteins, such as β-1,3-glucanases, and chitinases, which degrade fungal cell walls and inhibit pathogen growth (Perumal et al., 2017). Chen et al. (2018) noted that hairy fig extracts enhanced the antifungal properties of chitosan-based coatings by boosting the activity of defense-related enzymes, such as chitinase and β-1,3-glucanase, in treated novel oranges.

### 4.2 Maintenance of postharvest quality (i.e., firmness, color, taste)

The postharvest application of extracts derived from F&V waste can maintain fresh produce texture, appearance, flavor, and taste during postharvest storage (Table 1, Table 2). Firmness of fresh produce is an important sensory characteristic that determines fresh produce commercial quality. During postharvest storage, there is a reduction in firmness of fresh produce as a result of ripening and senescence (Payasi et al., 2009; Sila et al., 2008). The firmness of fresh F&V is linked to their cell wall composition and turgor pressure. Various cell wall modifying enzymes are involved in the degradation of cell wall polysaccharides resulting in fresh produce softening (Sila et al., 2008). ROS are also involved in fresh produce softening by accelerating the degradation of cell walls (Wei et al., 2025). Studies have noted that the postharvest application of extracts from F&V waste either incorporated into edible coatings or applied as a solution can contribute to the maintenance of fresh produce firmness during storage (Table 1, Table 2). Various mechanisms can be involved in firmness maintenance. For instance, natural extracts can promote the antioxidant system (antioxidant enzymes and antioxidant compounds) in the treated F&V, which deactivates ROS (i.e., OH^●^, H_2_O_2_, etc.), resulting in the reduction of lipid peroxidation of cell membranes and prevention of cell wall degradation (Fig. 3). For instance, Pontes et al. (2023) noted that the incorporation of cashew extract into xyloglucan-based coating maintained guava firmness by enhancing the activity of antioxidant enzymes such as SOD, catalase (CAT), and ascorbate peroxidase (APX), as well as increasing the synthesis of antioxidant compounds. Similar results reported by Chen et al. (2018) in navel oranges coated with chitosan-based coatings incorporated with hairy fig extracts. Another potential mechanism of natural extracts is the direct inactivation of enzymes involved in the degradation of cell wall polysaccharides in treated F&V. For instance, Megha et al. (2021) reported that incorporating pomegranate peel extract into a chitosan-based coating helped to maintain pear firmness by reducing the activity of key cell wall-degrading enzymes, including polygalacturonase (PG), pectin methylesterase (PME), and cellulase. Similar findings were reported by other studies who investigated the effects of edible films enriched with extracts from F&V waste (Pontes et al., 2023). Comparable results have also been observed when extracts are added into packaging materials. For instance, Hu et al. (2022) attributed the improved firmness of bananas packaged in chitosan-based films containing persimmon peel extract to reduced activity of the same hydrolytic enzymes mentioned above. The incorporation of natural extracts into coating and packaging materials, or their application as a solution, can reduce water loss and help maintain turgor pressure by improving the water vapor barrier properties. Further research is needed to elucidate the mechanisms by which natural extracts prevent water loss in fresh F&V.

Appearance is one of the most important sensory attributes of fresh produce, significantly influencing consumers' initial purchasing decisions. Among the visual qualities, color is considered the most critical component. The application of extracts derived from F&V waste has shown potential in preserving color during postharvest storage, whether incorporated into edible coatings and packaging materials or applied directly as a solution (Table 1, Table 2). However, the exact mechanisms by which these natural extracts maintain color remain underexplored. One possible indirect mechanism involves the prevention of pigment degradation through the enhancement of the antioxidant system in fresh produce, which helps inactivate excess ROS. ROS have been implicated in the degradation of key pigments such as chlorophylls, carotenoids, and anthocyanins (Amorim et al., 2023; Cerqueira et al., 2023; Semitsoglou-Tsiapou et al., 2022; Wahab et al., 2022). In addition, enzymatic browning is an undesirable reaction in fresh produce, negatively impacting its marketability. Studies have demonstrated that extracts from F&V waste can mitigate browning by reducing the activity of polyphenol oxidase (PPO), a copper-containing oxidoreductase that catalyzes the oxidation of polyphenols into quinones—intermediates in the formation of brown pigments (e.g., melanin) (Hu et al., 2022; Papoutsis & Edelenbos, 2021; Tinello & Lante, 2018; Wahab et al., 2022). Hu et al. (2022) noted reduced PPO activity in bananas packaged in chitosan-based film enriched in persimmon peel extract.

Respiration is closely linked to the storability of fresh produce, as higher respiration rates are generally associated with shorter shelf-life. Several studies have demonstrated that the postharvest application of natural extracts derived from F&V waste can reduce the respiration rate of treated produce during storage (Damas-Job et al., 2023; Kumar, Pratibha, Neeraj, Ojha, et al., 2021; Riaz et al., 2021; Zhang et al., 2020). However, the exact mechanism by which these natural extracts influence respiration remains unclear. A reduction in respiration rate may help explain the observed maintenance of taste and flavor in treated produce, as respiration consumes key metabolic compounds such as sugars and organic acids, which are essential to flavor quality. These compounds are used as substrates in energy production and the synthesis of secondary metabolites. Therefore, slowing respiration through natural extract treatments may contribute to flavor preservation by conserving these critical compounds during storage.

## 5 Challenges and future perspectives

The valorization of F&V waste for bioactive compound extraction presents a sustainable approach to reducing postharvest losses and promoting circular economy principles. However, despite its potential, several challenges hinder large-scale implementation. Addressing these barriers requires technological advancements, regulatory adaptations, and interdisciplinary research. This section highlights the key challenges and outlines future directions to optimize the use of F&V waste-derived bioactive compounds.

### 5.1 Challenges

#### 5.1.1 Extraction efficiency and scalability constraints

One of the major challenges in utilizing bioactive compounds from F&V waste is the efficiency and scalability of extraction methods. Traditional solvent-based extraction techniques, such as Soxhlet and maceration, are widely used but have environmental and economic drawbacks due to high solvent consumption and energy demands (Gallego et al., 2021). Alternative green extraction methods including ultrasound-assisted extraction, supercritical fluid extraction, and enzyme-assisted extraction offer more sustainable solutions but remain costly and complex to scale up for industrial applications (Chemat et al., 2019). Additionally, variations in bioactive compound yield due to differences in plant variety, harvest conditions, sample pretreatment (i.e., drying methods and conditions), and storage parameters pose further challenges in ensuring consistency and reproducibility in extraction processes (Freitas et al., 2021; Papoutsis et al., 2017). Indeed, there is sufficient evidence supporting the potential of extracts derived from F&V waste for use in postharvest handling systems. However, most studies focus primarily on extending the postharvest quality of fresh produce, without addressing production costs or system profitability.

#### 5.1.2 Lack of standardization and compound characterization

While several studies have demonstrated the positive effects of crude extracts on postharvest physiology, most have not characterized the specific bioactive compounds responsible for these effects. Understanding the precise mechanisms of action of these extracts is essential for optimizing their functionality and enhancing their commercial viability. Furthermore, the lack of standardized guidelines for the formulation and application of bioactive extracts limits their widespread use in postharvest treatments (Szabo et al., 2025).

#### 5.1.3 Regulatory and safety concerns

Stringent food safety regulations govern the use of bioactive compounds in food applications, posing a significant barrier to commercialization. Regulatory agencies such as the European Food Safety Authority (EFSA) and the U.S. Food and Drug Administration (FDA) require extensive testing to ensure that these compounds meet safety standards before they can be incorporated into food systems (EFSA et al., 2021). Potential concerns regarding pesticide residues, heavy metals, and microbial contaminants in agricultural waste necessitate rigorous screening, further complicating regulatory approval processes (Sagar et al., 2018). Establishing clear guidelines and safety protocols will be essential for the successful adoption of bioactive extracts in food preservation.

#### 5.1.4 Challenges in application and consumer acceptance

The practical application of bioactive compounds to fresh produce poses additional challenges. Optimizing extract concentration is crucial to maintain the sensory attributes, nutritional quality, and safety of treated products. Furthermore, the method of application whether through edible coatings, dipping solutions, or packaging integration must be carefully evaluated to maximize efficiency while minimizing undesirable effects (Martins et al., 2024). Consumer acceptance also plays a pivotal role, as skepticism regarding the use of waste-derived ingredients in food products may hinder market penetration. Effective communication strategies are necessary to raise awareness about the benefits of bioactive extracts in improving food quality and sustainability.

### 5.2 Future perspectives

#### 5.2.1 Advancements in green extraction technologies

To overcome extraction challenges, future research should focus on developing cost-effective, scalable, as well as environmentally and human-friendly extraction techniques. The use of non-toxic, biodegradable solvents should be prioritized to ensure food safety and environmental sustainability. Furthermore, emerging encapsulation technologies such as nanoemulsions, liposomes, and microencapsulation can enhance the stability, bioavailability, and controlled release of bioactive compounds (Rezagholizade-Shirvan et al., 2024). Integrating artificial intelligence (AI) and machine learning in process optimization may also help improve extraction efficiency and predict compound stability in various food applications (Liu et al., 2025).

#### 5.2.2 Comprehensive characterization and mechanistic studies

Future studies should focus on identifying and characterizing the key bioactive compounds responsible for postharvest quality improvements. The application of combined-omics approaches (transcriptomics, proteomics, and metabolomics) will facilitate a deeper understanding of the molecular and biochemical changes occurring in treated fresh produce. Specifically, metabolomics will facilitate understanding the potential effects of natural compounds on metabolites involved in postharvest quality (sensory, nutritional, and commercial) of the treated F&V (Wahid et al., 2022; Yun et al., 2022). Proteomics will facilitate the detection of proteins related to F&V postharvest quality maintenance, and are synthesized as a response to the application of natural compounds (Mathabe et al., 2020; Shang et al., 2021). Similarly, transcriptomics will contribute novel knowledge related to RNA transcripts in the tissues of the F&V treated with natural extracts (Cheng et al., 2023; Shang et al., 2021). Shang et al. (2021) employed integrated proteomic and transcriptomic analyses to elucidate the potential antifungal mechanism of the indoloquinoline alkaloid neocryptolepine against *Rhizoctonia solani*. Additionally, research should explore the penetration mechanisms of bioactive extracts in F&V tissues when applied through different delivery methods, such as edible coatings, dipping solutions, or packaging integration. Investigating the individual and synergistic effects of compounds will further clarify their roles in the prevention of fresh produce spoilage.

#### 5.2.3 Enhancing antimicrobial and food safety applications

Given the increasing concerns over foodborne pathogens and mycotoxins, more studies should explore the antimicrobial properties of bioactive extracts from F&V waste. Investigations should focus on their potential to inhibit pathogens such as *Salmonella spp.*, *Escherichia coli*, and *Listeria monocytogenes* while preventing mycotoxin contamination. Recent studies have shown that plant-derived bioactive compounds can effectively inhibit fungal growth and mitigate mycotoxin production (Dwivedy et al., 2018; Jiang et al., 2022). Further research on these antimicrobial mechanisms will be essential for integrating bioactive extracts into food safety strategies.

#### 5.2.4 Integration into circular economy models and policy support

The sustainable utilization of bioactive compounds from F&V waste aligns with circular economy principles, where waste is repurposed into high-value products. To facilitate large-scale adoption, interdisciplinary collaborations between academia, industry, and policymakers are essential. Public-private partnerships can drive investment in waste valorization technologies, while supportive regulatory frameworks will streamline the approval process for bioactive extract applications (FAO, 2022; Kumar et al., 2022). Additionally, increasing consumer awareness and market-driven incentives will encourage industries to incorporate waste-derived bioactive compounds into food preservation strategies. Finally, future studies investigating the valorization of extracts from F&V waste as a postharvest treatment, are encouraged to evaluate the production costs and system profitability. This information will support the scalability of F&V waste valorization.

## 6 Conclusion

The utilization of bioactive compounds from F&V waste holds great potential for reducing postharvest losses, enhancing food safety, and promoting sustainability in the food industry. However, challenges related to extraction efficiency, standardization, regulatory constraints, and consumer acceptance must be addressed to facilitate large-scale implementation. Future research should focus on improving extraction technologies, characterizing bioactive compounds, and understanding their mechanisms of action. Moreover, integrating these compounds into circular economy models, enhancing policy support, and fostering industry collaborations will be critical for their commercial success. By addressing these research gaps and technological limitations, the valorization of F&V waste can transition from experimental studies to real-world applications, ultimately contributing to a more sustainable and resilient global food system.

## CRediT authorship contribution statement

**Konstantinos Papoutsis:** Writing – review & editing, Writing – original draft, Supervision, Conceptualization. **Zahra Shams:** Writing – original draft. **Mahboobeh Yazdani:** Writing – review & editing. **Faraz Muneer:** Writing – original draft. **Mahbubjon Rahmatov:** Writing – review & editing. **Nikwan Shariatipour:** Writing – original draft. **Evelyn Elizabeth Villanueva-Gutierrez:** Writing – review & editing. **Monalisa Sahoo:** Writing – review & editing, Writing – original draft.

## Declaration of competing interest

The authors declare that they have no known competing financial interests or personal relationships that could have appeared to influence the work reported in this paper.

## Data Availability

No data was used for the research described in the article.
